# A tissue-selective estrogen complex as treatment of osteoporosis in experimental lupus

**DOI:** 10.1177/09612033211067984

**Published:** 2022-01-21

**Authors:** Jauquline Nordqvist, Cecilia Engdahl, Julia M Scheffler, Priti Gupta, Karin L Gustafsson, Marie K Lagerquist, Hans Carlsten, Ulrika Islander

**Affiliations:** 1 Department of Rheumatology and Inflammation Research, 70712 University of Gothenburg Sahlgrenska Academy, Goteborg, Sweden; 2 Department of Internal Medicine and Clinical Nutrition, 70712 University of Gothenburg Sahlgrenska Academy, Goteborg, Sweden

**Keywords:** Systemic lupus erythematosus, osteoporosis, tissue-selective estrogen complex, selective estrogen receptor modulator, estradiol, MRL/lpr

## Abstract

Osteoporosis is a common secondary complication in patients with systemic lupus erythematosus (SLE). Current osteoporosis treatment with bisphosphonates has some negative side effects and there is a lack of data regarding newer treatments options for SLE associated osteoporosis. The tissue-selective estrogen complex (TSEC) containing conjugated estrogens and the selective estrogen receptor modulator bazedoxifene (Bza) is approved for treatment of postmenopausal vasomotor symptoms and prevention of osteoporosis. However, it has not been evaluated for treatment of osteoporosis in postmenopausal SLE patients. Ovariectomized MRL/*lpr* mice constitute a model for postmenopausal lupus that can be used for osteoporosis studies. We used this model in a set of experiments where the mice were treated with different doses of 17β-estradiol-3-benzoate (E2), Bza, or TSEC (E2 plus Bza), administered in the early or late phases of disease development. The skeleton was analyzed by dual-energy X-ray absorptiometry, peripheral quantitative computed tomography, and high-resolution microcomputed tomography. The lupus disease was assessed by determination of proteinuria, hematuria, and lupus disease markers in serum. Treatment with medium dose TSEC administered in early disease protected ovariectomized MRL/*lpr* mice from trabecular bone loss, while there were no differences in lupus disease parameters between treatments. This is the first experimental study to investigate TSEC as a potential new therapy for osteoporosis in postmenopausal SLE.

## Introduction

Systemic lupus erythematosus (SLE) is a chronic autoimmune disease characterized by inflammation and tissue destruction that can affect nearly every organ in the body. The inflammation process in patients with SLE is mediated by immune complex deposits followed by complement activation, which often affects the skin and kidneys. Biological sex plays a major role in SLE development, resulting in considerably higher prevalence in women.^1^ Estrogen influences several parts of the immune system including lymphocytes, antibody production, the complement- and interferon-systems, which are all important in SLE pathogenesis.^2,3^

One major secondary complication of SLE is osteoporosis and associated fractures. Osteopenia occurs already in pre-menopausal SLE patients but worsens significantly after menopause.^4^ Additionally, a recent publication showed lower BMD in pre-menopausal SLE patients compared with healthy controls,^5^ further highlighting the risk for osteoporosis development in these patients. SLE patients also have an almost 3-fold increased risk of vertebral fractures compared with age-matched controls.^6^ The reasons why patients with SLE develop osteoporosis is multifactorial where autoimmune inflammation, renal failure, hormonal factors, and glucocorticoid treatments can negatively impact bone.^7^ The skeleton is continuously remodeled by bone resorbing osteoclasts and bone forming osteoblasts. During osteoporosis, bone homeostasis is shifted toward bone resorption, which affects both cortical and trabecular bone. Another major cause of osteoporosis in women is menopause and SLE is associated with early menopause, adding additional risk for these patients to develop osteoporosis.^8,9^

Hormone replacement therapy (HRT) containing estrogen protects against osteoporosis and vasomotor symptoms (VMS) in postmenopausal women.^10^ But HRT is also associated with negative side effects including increased risk of invasive breast cancer.^11^ Previous studies describing the effects of HRT in patients with SLE are contradictive. For example, HRT has been shown to induce flares in SLE patients,^12^ while a short course randomized trial with HRT in SLE patients showed no increased risk of severe lupus flares and only a slight increased risk of mild to moderate flares.^13^

Selective estrogen receptor modulators (SERMs) are molecules that bind estrogen receptors (ER) and induce tissue-dependent agonistic or antagonistic effects. Thus, an optimal SERM would exhibit the protective effects of estrogen on the skeleton and VMS, but completely lack the negative side effects on breast- and endometrial tissues. There are several SERMs currently used in clinical practice. The second-generation SERM raloxifene is approved for treatment of postmenopausal osteoporosis^14^ and one study showed that treatment with raloxifene was protective against bone loss in postmenopausal SLE patients without increasing disease flares.^15^ The third-generation SERM bazedoxifene (Bza) is the first SERM that both counteracts postmenopausal osteoporosis and prevents non-vertebral fractures in postmenopausal women.^16^ A tissue-selective estrogen complex (TSEC) comprises estrogens combined with a SERM, where the SERM blocks the negative effects of estrogens.^17^ The combination of conjugated estrogens and Bza is the first TSEC approved by *FDA* and *EMA* for prevention of osteoporosis and treatment of VMS. However, neither Bza alone nor TSEC, have been evaluated as treatment of osteoporosis in postmenopausal SLE patients.

To date, there are no anti-osteoporotic drugs available on the indication secondary osteoporosis in SLE, neither for pre- nor postmenopausal patients. Therefore, studies to find new and safe treatment options are needed to avoid fractures in these patients in the future. There are several antiresorptive agents available for osteoporosis therapy, for example, bisphosphonates, denosumab, and raloxifene. Bisphosphonates are currently the first-line treatment for osteoporosis.^18^ However, bisphosphonates are contraindicated in fertile women^19^ and the adherence to oral treatment is relatively low.^20^ For denosumab, data are lacking regarding treatment of patients on immunosuppressive agents.^21^ Raloxifene as treatment of osteoporosis is debated as it has not been possible to show risk reduction for non-vertebral fractures, and because of the associated increased risk for thromboembolism.^22^

MRL/MpJ-*Fas*^*lpr*^/J (MRL/*lpr*) mice spontaneously develop SLE-like symptoms, including systemic autoimmunity, production of anti-double stranded (ds) DNA antibodies, and immune-complex driven inflammation in the kidney glomeruli.^23^ In a previous publication, we showed that ovariectomized (OVX) MRL/*lpr* mice display reduced cortical BMD compared with OVX congenic control mice (MRL/++) and constitute a valuable experimental model for studies of osteoporosis development in postmenopausal SLE.^24^ Treatment of MRL/*lpr* mice with pharmacological doses of estrogen worsens the lupus disease.^25^ On the contrary, the first-generation SERM tamoxifen was shown to ameliorate disease symptoms in MRL/*lpr* mice,^26^ and treatment of NZB/W F1 lupus-prone mice with the combination of 17β-estradiol-3-benzoate (E2) plus raloxifene, reduced kidney damage and serum levels of anti-dsDNA antibodies compared with E2-treatment alone.^27^ We have previously shown that treatment with Bza significantly increases total BMD in healthy OVX mice,^28^ and that treatment with the TSEC (E2 plus Bza) protects against both cortical and trabecular bone loss in mice subjected to experimental arthritis.^29^ To our knowledge, the effects of treatment with Bza or TSEC have not previously been investigated in experimental postmenopausal lupus.

In this study, OVX MRL/*lpr* mice were treated with TSEC comprising E2 combined with Bza, and effects on the skeleton were evaluated. The study was conducted using different dose combinations of E2 and Bza, and treatments were initiated at either the early or late stages of disease development. Results show that treatment with TSEC, as well as Bza alone, protects against bone loss in experimental postmenopausal lupus, while the levels of proteinuria and hematuria remained unchanged.

## Materials and methods

### Mice

All work was approved by the ethical review board in Gothenburg. Female MRL/MpJ-*Fas*^*lpr*^/J (MRL/*lpr*) mice were purchased from Jackson Laboratory (Bar Harbor, ME, USA). They were kept in groups of 3–10 mice per cage under controlled environmental conditions and fed soy-free chow and tap water *ad libitum*.

### Proteinuria and hematuria

Mice were weighed and controlled for proteinuria and hematuria using dipsticks (Multistix®7, Siemens Healthcare Diagnostics Inc., Tarrytown, USA) once every week up to 10 weeks and twice weekly thereafter. Proteinuria and hematuria were scored and quantified according to the manufacturers’ instructions. Mice that were deceased, or terminated for ethical reasons (>10% weight loss in 1 week) prior to the experiment end-point, were included in the analyses of proteinuria and hematuria.

### Ovariectomy (OVX)

OVX was performed under Isoflurane (Baxter Healthcare Corporation, Chicago, IL, USA) anesthesia by a midline incision of the skin, bilateral incisions of the peritoneum, and removal of the ovaries. The peritoneum was closed with sutures and the skin with metallic clips. Sham surgery included all steps except removal of the ovaries. The mice were given subcutaneous (s.c.) injections of Carprofen (Rimadyl, Orion Pharma Animal Health, Sollentuna, Sweden) as post-operative analgesia.

### Treatments

MRL/*lpr* mice were OVX or sham operated and treated with s. c. injections of vehicle (Veh) miglyol oil (Vendico Chemical AB, Malmo, Sweden), 17β-estradiol-3-benzoate (E2, Sigma Aldrich, St Louis, MO, USA), bazedoxifene (Bza, Pfizer Inc., NY, USA), or a tissue-selective estrogen complex (TSEC: E2 + Bza). Treatments were given 5 days per week for 3 weeks in various dose combinations and administered at different time points in the disease development:

Low dose treatment in active disease: 0.1 μg E2 + 24 μg Bza/mouse/day. The initial number of mice was 10 per OVX group and five in the sham group. Two mice in OVX Veh, two in OVX Bza, and one in the OVX TSEC group were deceased or terminated for ethical reasons prior to the experiment end-point. These mice were included in the analysis of proteinuria and hematuria.

Medium dose treatment in early disease: 0.15 μg E2 + 36 μg Bza/mouse/day. The initial number of mice was 10 per OVX group and five in the sham group. One mouse in the OVX Veh treated group deceased prior to the experiment end-point but was included in the analysis of proteinuria and hematuria. One mouse in the OVX Veh group was removed from all analyses due to unsuccessful OVX.

High dose treatment in early disease: 0.5 μg E2+ 48 μg Bza/mouse/day. The initial number of mice was 11 per OVX group and six in the sham group. One mouse in the OVX Veh group was removed from all analyses due to unsuccessful OVX.

### Tissue collection and preparation of cell suspensions

At termination of the experiments, blood was collected and sera stored at −20°C until use. Uteri were dissected and weighed. To obtain single cell suspensions from bone marrow (BM), one femur was dissected and BM flushed with PBS using a syringe. Erythrocytes were lysed using Tris-buffered 0.83% NH_4_Cl solution (pH 7.4) and the cells counted in an automated cell counter (Sysmex Europe GmbH, Norderstedt, Germany).

### Serum analyses

Serum levels of antibodies to double stranded (ds) DNA (total IgA, IgG, and IgM) (Mouse anti-dsDNA antibodies total Ig ELISA Kit, Alpha Diagnostic, San Antonio, USA), IgM (Mouse IgM ELISA kit, Bethyl Laboratories, Montgomery, USA), IgG (Mouse IgG ELISA quantitation set, Bethyl Laboratories), interleukin-6 (IL-6) (Mouse IL-6 ELISA Kit, Life Technologies), urea (colorimetric Urea Assay Kit, Abcam, Cambridge, United Kingdom), C-terminal type I collagen (CTX-I, ELISA RatLaps kit, Immunodiagostic Systems, Boldon, UK), procollagen type I N propeptide (PINP, Immunodiagostic Systems), and parathyroid hormone (PTH, Elabscience, Houston, USA) were analyzed according to the manufacturers’ instructions.

### Bone analyses

#### Dual-energy X-ray absorptiometry (DXA)

Total body areal bone mineral density (aBMD) and lumbar spine (L3-L6) aBMD were analyzed once or twice during the experiments in isoflurane-anesthetized mice, and at termination of the experiments, using DXA (Lunar PIXImus densitometer, Wipro GE Healthcare).

### Peripheral quantitative computed tomography (pQCT)

At termination of the experiments one femur and lumbar vertebra (L5) were fixed in 4% formaldehyde. Femur BMD was defined by Stratec pQCT XCT Research M (software version 5.4; Norland, Fort Aktison, USA) at 70 μm resolution, as previously described.^30^ In short, mid-diaphyseal pQCT scans were performed for measuring cortical bone. Metaphyseal pQCT scans were performed for measuring trabecular bone, which was defined by setting an inner area to 45% of the total cross-sectional area.

### High-resolution microcomputed tomography (μCT)

Femur and L5 were analyzed using the high-resolution 1172 µCT (Bruker MicroCT, Aartselaar, Belgium) as described by Wu and colleagues.^31^ In brief, trabecular bone in femur was measured 560 μm from the proximal growth plate extending 134.5 μm in the proximal direction and cortical bone in femur was measured starting at 5.37 mm from the growth plate and extending 134.5 μm in the proximal direction. The trabecular bone of L5 was determined (excluding cortical bone) starting at 4.5 μm caudal of the pedicles and measured 157.5 μm in the caudal direction.

### Statistical analysis

Statistical analyses were performed using GraphPad Prism (Graph Pad Software Inc., La Jolla, USA). Groups were compared using one-way ANOVA followed by Sidak’s post-hoc analysis. Sham operated mice were only compared with Veh treated OVX mice, while all OVX groups independent of treatments were compared against each other. Results are presented as mean ± standard error of the mean (SEM). Kaplan–Meier curves for proteinuria and hematuria versus time were analyzed using Log-Rank test. A *p*-value of *p* < 0.05 was considered statistically significant.

## Results

The aim of this study was to investigate if the TSEC—E2 combined with Bza—can be used as treatment to prevent the development of osteoporosis in experimental postmenopausal lupus using OVX MRL/*lpr* mice.

### Treatment with medium dose TSEC in the early phase of lupus

**Figure 1.**
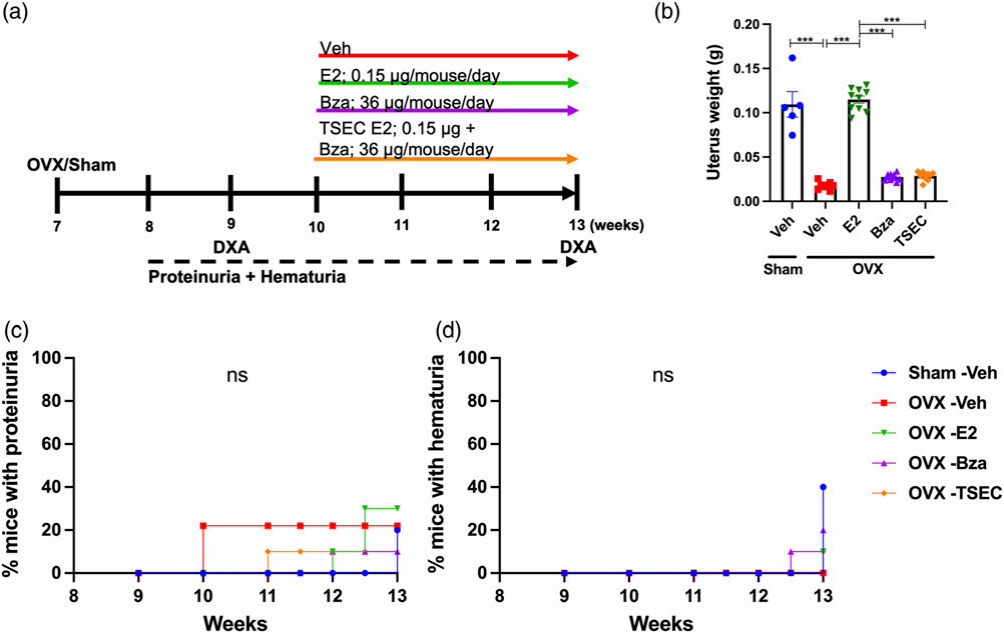
**Treatment with medium dose TSEC during early disease does not influence the degree of proteinurea and hematuria.** Ovariectomized (OVX) or sham operated MRL*/lpr* mice were treated 5 days per week for 3 weeks with medium doses of estrogen (E2; 0.15 μg/mouse/day), bazedoxifene (Bza; 36 μg/mouse/day), tissue-selective estrogen complex (TSEC; E2+Bza), or vehicle (Veh), in the early stage of lupus disease development (mouse age 10 weeks at treatment start). a Schematic overview of the experimental setup. b Uterus weights, presented as mean+/− SEM. Frequency of mice with c proteinuria and d hematuria. n = 5−10 mice per group. One-way ANOVA followed by Sidak’s post-hoc test was used in (b). Log rank test was used in (**c**) and (d). ***p < 0.001, ns = not significant.

MRL/*lpr* mice were subjected to OVX or sham surgery at 7 weeks of age and treated with medium dose E2, Bza, TSEC, or Veh (Figure 1(a)). Total mouse weights did not differ between groups at termination (data not shown). As expected, OVX surgery reduced the uterus weight compared with sham, while treatment of OVX mice with E2 restored the uterus weight to sham level. Mice treated with Bza or TSEC had uterus weights comparable with OVX Veh (Figure 1(b)).

The degree of proteinuria and hematuria did not differ between treatment groups (Figure 1(c) and (d)) and serum analyses revealed no differences in the levels of anti-dsDNA antibodies, urea, IL-6, IgM, or IgG between the groups (Supplementary Table 1).

The effects of TSEC treatment on the trabecular (Tb.) and cortical (Ct.) bone compartments were evaluated in detail using DXA, pQCT and μCT (methods that represent increasing levels of resolution).

**Figure 2.**
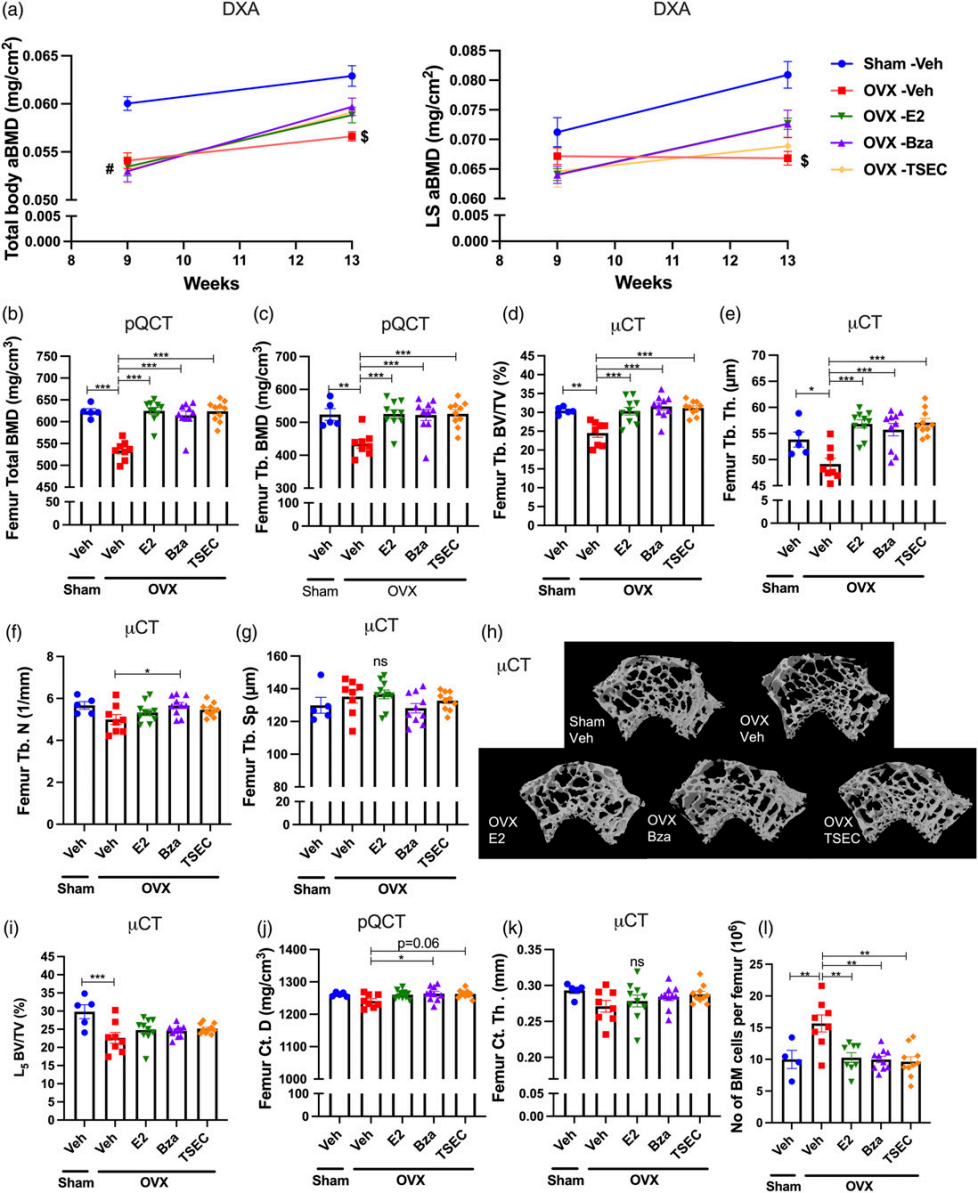
**Treatment with medium dose TSEC in early disease protects against trabecular but not cortical bone loss.** Ovariectomized (OVX) or sham operated MRL*/lpr* mice were treated 5 days per week for 3 weeks with medium doses of estrogen (E2; 0.15 μg/mouse/day), bazedoxifene (Bza; 36 μg/mouse/day), tissue-selective estrogen complex (TSEC; E2+Bza) or vehicle (Veh), in the early stage of lupus disease development (mouse age 10 weeks at treatment start). Total and trabecular bone parameters were defined. a Total body and lumbar spine (LS) areal bone mineral density (aBMD) determined by dual-energy X-ray absorptiometry (DXA) at 9 and 13 weeks of age. b Total femur bone mineral density (BMD) and c trabecular (Tb.) BMD determined by peripheral quantitative computed tomography (pQCT) at termination. d Bone volume/total volume (BV/TV), e Tb. thickness (Th.), f Tb. number (N), and g Tb. separation (Sp.) determined with microcomputed tomography (μCT) at termination. h Representative μCT images of femur Tb. bone. i BV/TV of lumbar vertebra 5 (L5) determined with μCT. Cortical bone parameters were defined. j Femur cortical (Ct.) density (D) measured with pQCT and k femur Ct. Th. measured with μCT. l Femur bone marrow (BM) cellularity. Data are expressed as mean+/- SEM *n* = 5−10 mice per group. One-way ANOVA followed by Sidak’s post-hoc test *p < 0.05, **p < 0.01, ***p < 0.001, ns = not significant. In (**a**) differences between sham Veh and all OVX groups are denoted by #, #*p* < 0.05. Differences between sham Veh and OVX Veh are denoted by $, $*p* < 0.05.

Total body areal BMD (aBMD) (Figure 2(a) left) and lumbar spine (LS) aBMD (Figure 2(a) right) were measured with DXA before the start of treatments and at termination of the experiment. All OVX groups (irrespective of intended treatment) had lower total body aBMD compared with sham at 9 weeks of age (Figure 2(a), left). At 13 weeks, the OVX Veh group had lower total body aBMD compared with sham, while OVX mice treated with E2, Bza, and TSEC had a tendency to higher total body aBMD compared with OVX Veh. For LS aBMD, the same alteration patterns were displayed, although statistically significant difference was only shown at 13 weeks for sham versus OVX Veh (Figure 2(a), right).

Trabecular bone parameters were evaluated using pQCT- (Figure 2(b) and (c)), and μCT analyses of femur (Figure 2(d)–(h)) and L5 vertebra (Figure 2(i)). As expected, OVX resulted in decreased BMD compared with sham operated mice (Figure 2(b)-(c)). The pQCT analysis revealed significantly higher total and trabecular BMD in femur from mice treated with E2, Bza, or TSEC compared with Veh (Figure 2(b)-(c)). The increase in trabecular bone was confirmed using micro-CT where the trabecular bone volume over total volume (BV/TV) was significantly increased by all treatments (Figure 2(d)). The same pattern was shown for trabecular thickness (Th) (Figure 2(e)), while for trabecular number (N) only Bza treatment resulted in a significant increase compared with OVX Veh (Figure 2(f)). No differences in trabecular separation (Sp) were detected between groups (Figure 2(g)). Figure 2(h) shows representative μCT images of femur trabecular bone. Analysis of the L5 vertebrae revealed a significant decrease in BV/TV after OVX, while no differences were displayed in response to treatments (Figure 2(i)).

Cortical (Ct.) bone parameters were evaluated using pQCT (Figure 2(j)) and μCT (Figure 2(k)) of femur. pQCT analysis revealed decreased cortical thickness after OVX, while no differences were shown for cortical thickness or content in response to treatments (data not shown). However, cortical density (D) was significantly increased by treatment with Bza (Figure 2(j)). μCT analysis showed no differences in cortical thickness between groups (Figure 2(k)). Serum analyses revealed no differences between the groups in markers of bone formation, procollagen type I N propeptide (PINP), or bone metabolism, parathyroid hormone (PTH), while the bone resorption marker C-terminal type I collagen (CTX-1) was significantly increased after OVX compared with sham (Supplementary Table 1).

Analyses of bone marrow (BM) cellularity showed that Veh treated OVX MRL/*lpr* mice had increased BM cellularity compared with sham, while this was suppressed by all treatments (Figure 2(l)).

### Treatment with high dose TSEC in the early phase of lupus

**Figure 3.**
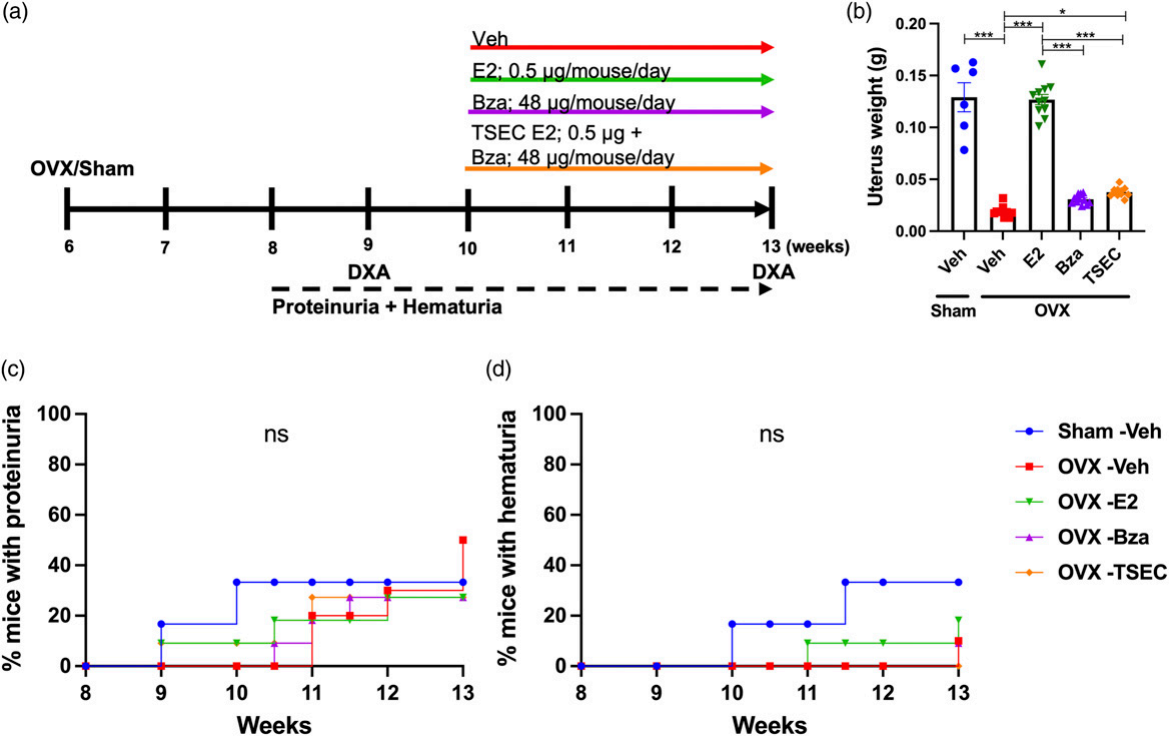
**Treatment with high dose TSEC in early disease leads to increased uterus weight, but does not influence the degree of proteinurea and hematuria.** Ovariectomized (OVX) or sham operated MRL*/lpr* mice were treated 5 days per week for 3 weeks with high doses of estrogen (E2; 0.5 μg/mouse/day), bazedoxifene (Bza; 48 μg/mouse/day), tissue-selective estrogen complex (TSEC; E2+Bza) or vehicle (Veh), in the early stage of lupus disease development (mouse age 10 weeks at treatment start). a Schematic overview of the experimental setup. b Uterus weights presented as mean+/- SEM. Frequency of mice with **c** proteinuria and d hematuria. n=6–11 mice per group. One-way ANOVA followed by Sidak’s post-hoc test was used in (b). Log-Rank test was used in (**c**) and (d). *p < 0.05, ***p < 0.001, ns = not significant.

To determine if increased doses of E2 and Bza would improve the protective effects of TSEC on cortical bone, MRL/*lpr* mice were subjected to OVX or sham surgery at 6 weeks of age and treated with high dose E2, Bza, TSEC, or Veh (Figure 3(a)). Total mouse weights did not differ between groups at termination (data not shown). OVX surgery reduced the uterus weight compared with sham, while E2 treatment restored it (Figure 3(b)). Treatment with Bza resulted in uterus weights similar to Veh, while there was a slight, but significant, increase in uterus weight after TSEC treatment (Figure 3(b)). This indicates that Bza was not able to fully antagonize the high dose of E2 in the TSEC treatment.

Proteinuria and hematuria did not differ between treatment groups (Figures 3(c) and (d)) and analyses of serum taken at termination of the experiment revealed no differences in the levels of anti-dsDNA antibodies, urea, IL-6, or IgG (Supplementary Table 2). The levels of IgM in serum were significantly increased after treatment with E2 compared with Veh, but not after treatment with Bza or TSEC (Supplementary Table 2).

**Figure 4.**
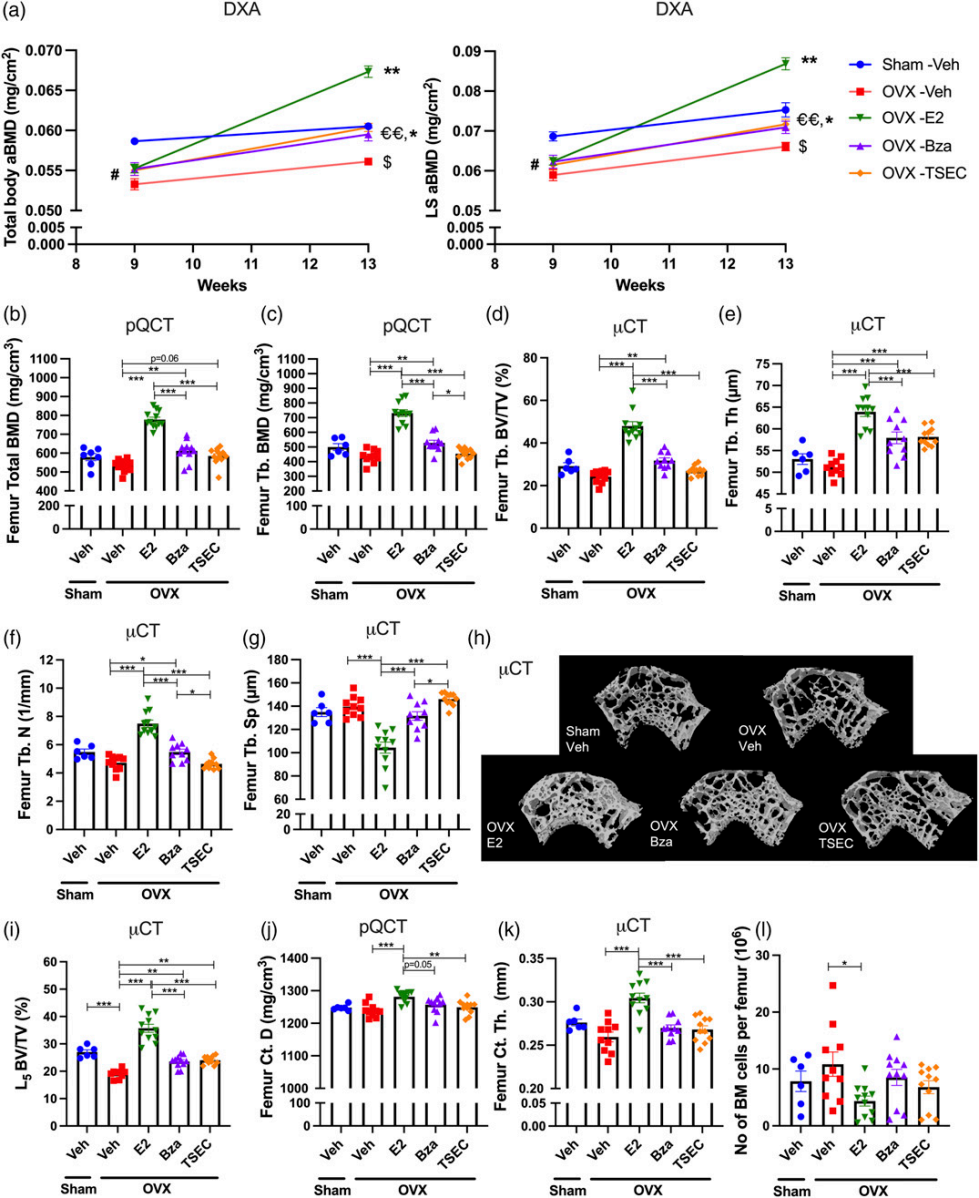
**Treatment with high dose TSEC in early disease does not influence cortical bone parameters.** Ovariectomized (OVX) or sham operated MRL*/lpr* mice were treated 5 days per week for 3 weeks with high doses of estrogen (E2; 0.5 μg/mouse/day), bazedoxifene (Bza; 48 μg/mouse/day), tissue-selective estrogen complex (TSEC; E2+Bza) or vehicle (Veh), in the early stage of lupus disease development (mouse age 10 weeks at treatment start). Total and trabecular bone parameters were defined. a Total body and lumbar spine (LS) areal bone mineral density (aBMD) measured by dual-energy X-ray absorptiometry (DXA) at 9 and 13 weeks of age. b Total femur bone mineral density (BMD) and **c** trabecular (Tb.) BMD determined by peripheral quantitative computed tomography (pQCT) at termination. d Bone volume/total volume (BV/TV), **e** Tb. thickness (Th), f Tb. number (*N*), and g Tb. separation (Sp) determined with microcomputed tomography (μCT) at termination. h Representative μCT image of femur Tb. bone. i BV/TV of lumbar vertebra 5 (L5) defined with μCT. Cortical bone parameters were defined. j Femur cortical (Ct.) density (D) measured with pQCT and k femur Ct. Th. measured with μCT. l Femur bone marrow (BM) cellularity. Data are expressed as mean+/-SEM. *n* = 6–11 mice per group. One-way ANOVA followed by Sidak’s post-hoc test *p < 0.05, **p < 0.01, ***p < 0.001. In (a), differences between sham Veh and all OVX groups are denoted by #. #*p* < 0.05. Differences between sham Veh and OVX Veh are denoted by $. $*p* < 0.05. Differences between OVX Veh and OVX E2, Bza or TSEC groups are denoted by *. *p < 0.05, **p < 0.01. Differences between OVX E2 and OVX Bza or OVX TSEC groups are denoted by €. €€*p* < 0.01.

The effects of TSEC treatment on the trabecular and cortical bone compartments were evaluated in detail using DXA, pQCT, and μCT. Results from the DXA scan showed that all OVX mice had a lower total body (Figure 4(a), left) and LS aBMD (Figure 4(a), right) before treatment start compared with sham, which sustained until termination for sham versus OVX Veh. Three weeks of treatment with high dose E2, Bza, or TSEC resulted in increased total body and LS aBMD in all treatment groups compared with OVX Veh. However, treatment with E2 alone resulted in more pronounced increase in both total body and LS aBMD compared with Bza and TSEC treatments (Figure 4(a)).

Trabecular bone parameters were further evaluated using pQCT- (Figure 4(b) and (c)), and μCT analyses of femur (Figure 4(d)–(h)) and L5 vertebra (Figure 4(i)). Surprisingly, OVX surgery only resulted in a non-significant trend towards decreased appendicular trabecular bone compared with sham (Figure 4(b)–(h)). pQCT analysis revealed that treatment with high dose E2 or Bza significantly increased total and trabecular BMD (Figure 4(b)-(c)), while only a non-significant trend towards increased total BMD was shown after treatment with TSEC compared with Veh (Figure 4(b)). The results were confirmed using micro-CT analysis where BV/TV (Figure 4(d)), trabecular thickness (Figure 4(e)) and trabecular number (Figure 4(f)) were significantly increased after treatment with E2 or Bza. E2 treatment also resulted in significantly decreased trabecular separation (Figure 4(g)). TSEC treatment only increased the trabecular thickness compared with Veh (Figure 4(e)). Figure 4(h) shows representative μCT images of femur trabecular bone. Analysis of L5 vertebrae revealed significantly decreased BV/TV after OVX and all treatments significantly increased BV/TV compared with OVX Veh (Figure 4(i)).

Cortical bone was evaluated using pQCT (Figure 4(j)) and μCT analyses (Figure 4(k)) of femur. Treatment with E2 caused a significant increase in cortical parameters compared with Veh, while no differences were detected after treatment with Bza or TSEC (Figure 4(j) and (k)). Serum analyses revealed no differences in CTX-1, PINP, or PTH, between groups (Supplementary Table 2).

BM cellularity in femur was reduced after treatment with E2 compared with Veh (Fig. 4l), while neither Bza nor TSEC treatment affected the BM cellularity.

### Treatment with low dose TSEC in established lupus

**Figure 5.**
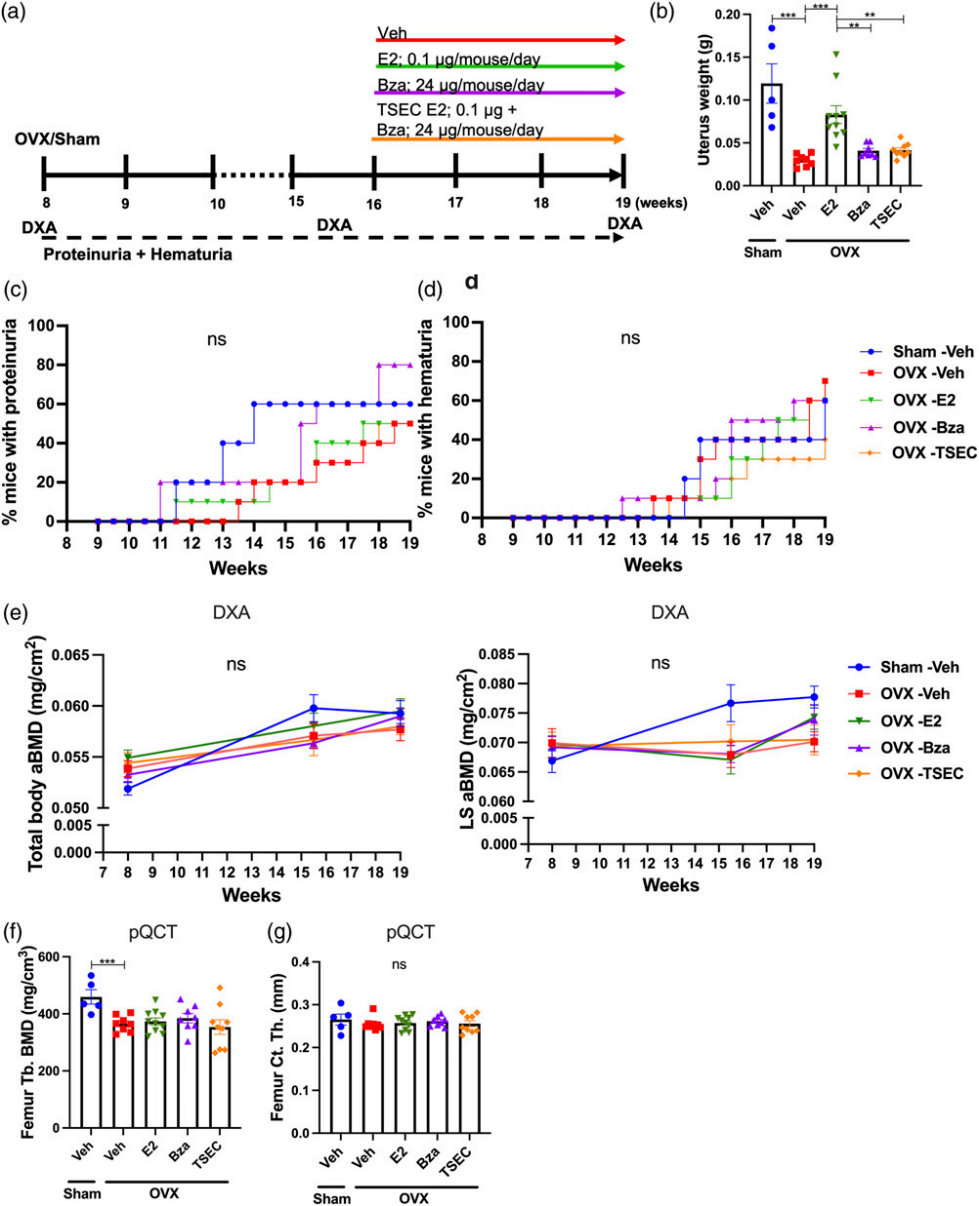
**Treatment with low dose TSEC in active disease does not influence the degree of proteinurea and hematurea, and does not protect against trabecular bone loss.** Ovariectomized (OVX) or sham operated MRL*/lpr* mice were treated 5 days per week for 3 weeks with low doses of estrogen (E2; 0.1 μg/mouse/day), bazedoxifene (Bza; 24 μg/mouse/day), tissue-selective estrogen complex (TSEC; E2+Bza) or vehicle (Veh), in a stage of active lupus disease development (mouse age 16 weeks at treatment start). a Schematic overview of the experimental setup. b Uterus weights presented as mean+/− SEM. Frequency of mice with **c** proteinuria and d hematuria. e Total body and lumbar spine (LS) areal bone mineral density (aBMD) determined by dual-energy X-ray absorptiometry (DXA) at 8, 15.5 and 19 weeks of age. f Femur trabecular (Tb.) bone mineral density (BMD) and g cortical (Ct.) thickness (Th.) analyzed with peripheral quantitative computed tomography (pQCT) at termination. Data are expressed as mean+/−SEM. *n*=5–10 mice per group. One-way ANOVA followed by Sidak’s post hoc test was used in (b and e-g). Log-Rank test was used in (**c**) and (d). **p < 0.01. ***p < 0.001, ns = not significant.

We have previously shown that treatment with low dose TSEC (0.1 μg E2 + 24 μg Bza/mouse/day) protects against cortical and trabecular bone loss in mice with experimental arthritis.^29^ Therefore, as an initial experiment for this study, we treated OVX MRL*/lpr* mice with low dose TSEC in the established phase of lupus (Figure 5(a)). Total mouse weights did not differ between groups at termination (data not shown). As expected, the uterus weight of OVX Veh mice was significantly lower compared with sham (Figure 5(b)). Treatment with low dose E2 increased the uterus weight, while low doses Bza or TSEC resulted in uterus weights similar to the OVX Veh group (Figure 5(b)).

Proteinuria and hematuria did not differ between groups (Figure 5(c) and (d)). Also, serum taken at termination of the experiment revealed no differences in the levels of anti-dsDNA antibodies, urea, IL-6, IgM, or IgG between groups (data not shown).

DXA analyses were performed at the time of OVX or sham surgery, before the start of treatments, and at termination of the experiment. The results showed no differences in total body and LS aBMD between the groups at any time-point (Figure 5(e)). pQCT analysis of femur revealed decreased trabecular BMD in OVX Veh mice compared with sham, but no differences between the different groups in either trabecular BMD or cortical thickness (Figure 5(f)-(g)). Furthermore, serum levels of CTX-1, PINP, and PTH were unchanged between groups (data not shown).

## Discussion

In this study we show that treatment with a TSEC (comprising E2 combined with Bza), protects against bone loss in a model of experimental postmenopausal lupus, without affecting the development of proteinuria and hematuria.

Menopause is a major cause for osteoporosis and ovariectomy (OVX) of mice is a well-established model to study both cortical and trabecular bone loss in postmenopausal osteoporosis. The MRL/*lpr* mouse strain spontaneously develops a lupus-like syndrome at young age, and in a previous study we confirmed that OVX MRL/*lpr* mice represents a valuable model for studies of osteoporosis development in postmenopausal lupus.^24^ In that study, bone deteriorating effects were primarily shown for cortical bone,^24^ which corresponds with the finding that cortical bone is predominantly affected in patients with SLE,^32^ although also trabecular bone has been shown to be affected.^33,34^

The protective effect of estrogen on the skeleton is well documented,^35^ but the effects of estrogen on the lupus disease development are contradictive. For example, SLE patients are at high risk for disease flares during pregnancy,^36^ while oral contraceptives are well tolerated in patients with stable disease,^37^ and the risk of HRT to induce flares in SLE patients varies greatly between studies.^12,13^ In experimental lupus models, treatment with a pharmacological dose E2 exacerbates the disease development,^25^ while tamoxifen, or the combination of E2 and raloxifene ameliorate disease symptoms.^26,27^ More studies are needed to fully elucidate the cellular and molecular mechanisms induced by estrogens, SERM and TSEC in lupus-prone mice.

In this study, we show that TSEC treatment results in protective effects on trabecular bone parameters. The relatively short treatment time is a possible explanation for why most effects are shown for trabecular bone. It remains to be determined whether protective effects could be reached also for cortical bone by longer treatment times. The doses used in this study are based on our previous publication where treatment with low dose TSEC was shown to protect against bone loss in experimental arthritis.^29^ In this study, low dose TSEC administered during active lupus disease did not protect against trabecular bone loss. Whether this was a result of too low treatment doses, or if the treatment was initiated at a time when the lupus disease was too severe, remains to be investigated. The doses we refer to as “medium” here are also relatively low, as displayed in OVX mice treated with E2, where the uterus weight is comparable to sham. Results show that osteoporosis in OVX MRL/*lpr* mice can be counteracted using the medium dose TSEC and Bza. With high treatment doses, E2 induced a substantial increase in bone mass, while Bza and TSEC failed to induce bone protective effects for several of the analyzed parameters. The high dose of E2 was also reflected by the uterus weights, where addition of Bza was unable to completely dampen the uteroproliferative effect of E2 in the TSEC treatment. Serum markers of bone remodeling and metabolism (CTX-1, PINP or PTH) were not affected by the treatments, irrespective of doses or time points for administration of the treatments. However, it is possible that differences in these markers could have been detected if serum samples had been taken earlier after initiation of treatments.

Development of proteinuria, hematuria and high levels of serum autoantibodies against dsDNA are common characteristics in SLE patients as well as in MRL/*lpr* mice. In this study we found no effects on these parameters in response to treatments. One limitation of this study is the lack of histological examinations of target organs including kidney, salivary glands and secondary lymphoid organs, which would provide further information regarding effects of treatments on lymphoproliferation, immune complex formations and glomerular damage. Thus, additional studies need to be conducted in order to elucidate the cellular and molecular mechanisms involved in the effects of TSEC treatment in postmenopausal lupus.

Osteopenia develops already in premenopausal SLE patients, although the condition drastically worsens after menopause.^4^ Hypothetically, TSEC treatment in postmenopausal SLE patients would not only be beneficial by protecting the skeleton and preventing fractures, it would also exert positive effects on VMS. A severe secondary complication of SLE is the increased risk for cardiovascular events.^38^ TSEC showed mostly favorable changes regarding the lipid profile in a pooled analysis of three randomized control trials.^39^ Furthermore, TSEC was shown to improve menopause-specific quality of life^40^ as well as daily minutes slept in menopausal women.^41^ However further studies are needed to elucidate the effects and safety profile of TSEC treatment in postmenopausal SLE.

In conclusion, in this study we found that treatment with medium dose TSEC given early in the disease protects from trabecular bone loss in OVX MRL/*lpr* mice while no differences in hematuria, proteinuria and immunoglobulin production could be detected in response to treatment. These findings encourage further studies using TSEC in both murine models and postmenopausal lupus patients.

## Supplemental Material

sj-pdf-1-lup-10.1177_09612033211067984 – Supplemental Material for A tissue-selective estrogen complex as treatment of osteoporosis in experimental lupus Click here for additional data file. Supplemental Material, sj-pdf-1-lup-10.1177_09612033211067984 for A tissue-selective estrogen complex as treatment of osteoporosis in experimental lupus by J Nordqvist, C Engdahl, JM Scheffler, P Gupta, KL Gustafsson, MK Lagerquist, H Carlsten and U Islander in Lupus
