# Supplementary material for: A tissue-selective estrogen complex as treatment of osteoporosis in experimental lupus
Source: Lupus. 2022 Jan 21;31(2):143–54. doi: 10.1177/09612033211067984 (PMC8832562; doi:10.1177/09612033211067984)
Supplement: sj-pdf-1-lup-10.1177_09612033211067984 – Supplemental Material for A tissue-selective estrogen complex as treatment of osteoporosis in experimental lupus [file sj-pdf-1-lup-10.1177_09612033211067984.pdf]

Supplementary table 1. Medium dose treatment in early disease. Serum analyses on week 13. Values shown as arithmetic mean ± SEM. Sham Veh vs. OVX Veh: * p<0.05.

	Sham Veh	OVX Veh	OVX E2	OVX Bza	OVX TSEC
**Anti-dsDNA** (kU/mL)	3181 ± 740	2225 ± 270	1911 ± 268	2153 ± 218	2564 ± 260
**Urea** (mg/dL)	75.53 ± 4.47	89.43 ± 4.11	123.92 ± 20.73	85.72 ± 6.48	103.42 ± 22.24
**IL-6** (pg/mL)	26.81 ± 14.16	82.44 ± 33.62	80.31 ± 47.91	168.09 ± 67.65	20.20 ± 7.07
**IgM** (mg/dL)	73.67 ± 7.32	75.86 ± 9.80	92.90 ± 9.09	68.30 ± 5.58	78.24 ± 7.17
**IgG** (mg/dL)	771.9 ± 316.8	784.2 ± 100.5	871.2 ± 257.4	911.9 ± 226.0	778.1 ± 171.2
**CTX-1** (ng/mL)	5.05 ± 0.98	16.05 ± 2.68*	12.51 ± 2.85	16.01 ± 1.56	8.40 ± 2.00
**PINP** (ng/mL)	9.73 ± 0.69	13.76 ± 4.51	8.75± 0.79	18.37± 9.00	8.45 ± 1.04
**PTH** (pg/mL)	11.28± 1.56	17.11 ± 2.23	20.22 ± 3.47	11.88 ± 1.01	13.41 ± 1.69

Anti-dsDNA - Antibodies to double stranded DNA; *IL-6* – interleukin 6; *Ig* – Immunoglobulin; *CTX-1* - C-terminal type I collagen; *PINP* - Procollagen type I N propeptide; *PTH* - Parathyroid hormone

Supplementary table 2. High dose treatment in early disease. Serum analyses on week 13. Values shown as arithmetic mean ± SEM. OVX Veh vs. OVX E2: *** p<0.001. OVX E2 vs. OVX Bza or OVX TSEC: ## p<0.01, ### p<0.001.

	Sham Veh	OVX Veh	OVX E2	OVX Bza	OVX TSEC
**Anti-dsDNA** (kU/mL)	4202 ± 1413	2828 ± 305	3058 ± 416	3612 ± 877	3255 ± 541
**Urea** (mg/dL)	94.46 ± 21.77	79.27 ± 4.26	75.94 ± 10.37	83.31 ± 5.16	78.51 ± 2.74
**IL-6** (pg/mL)	15.61 ± 6.03	32.24 ± 15.44	12.23 ± 7.03	29.69 ± 25.79	17.99 ± 6.96
**IgM** (mg/dL)	92.53 ± 8.26	74.86 ± 4.83	122.44± 9.02***	76.32 ± 7.12###	85.30 ± 6.26##
**IgG** (mg/dL)	391.3 ± 75.4	841.2 ± 132.8	718.9 ± 130.4	618.2 ± 101.6	626.0 ± 147.7
**CTX-1** (ng/mL)	15.06 ± 1.74	25.60 ± 1.90	23.55 ± 2.24	23.39 ± 1.36	20.83 ± 3.24
**PINP** (ng/mL)	7.13 ± 0.38	8.05 ± 0.98	10.17 ± 1.99	8.68 ± 1.57	8.56 ± 0.94
**PTH** (pg/mL)	32.50 ± 7.97	23.82 ± 5.38	21.61 ± 3.66	13.31 ± 1.21	21.13 ± 6.30

Anti-dsDNA - Antibodies to double stranded DNA; *IL-6* – interleukin 6; *Ig* – Immunoglobulin; *CTX-1* - C-terminal type I collagen; *PINP* - Procollagen type I N propeptide; *PTH* - Parathyroid hormone
